# Prediction of air pollutant concentrations based on TCN-BiLSTM-DMAttention with STL decomposition

**DOI:** 10.1038/s41598-023-31569-w

**Published:** 2023-03-22

**Authors:** Wenlin Li, Xuchu Jiang

**Affiliations:** School of Statistics and Mathematics, Zhongnan University of Economics and Law, Wuhan, 430073 China

**Keywords:** Climate sciences, Environmental sciences, Environmental social sciences, Engineering, Mathematics and computing

## Abstract

A model with high accuracy and strong generalization performance is conducive to preventing serious pollution incidents and improving the decision-making ability of urban planning. This paper proposes a new neural network structure based on seasonal–trend decomposition using locally weighted scatterplot smoothing (Loess) (STL) and a dependency matrix attention mechanism (DMAttention) based on cosine similarity to predict the concentration of air pollutants. This method uses STL for series decomposition, temporal convolution, a bidirectional long short-term memory network (TCN-BiLSTM) for feature learning of the decomposed series, and DMAttention for interdependent moment feature emphasizing. In this paper, the long short-term memory network (LSTM) and the gated recurrent unit network (GRU) are set as the baseline models to design experiments. At the same time, to test the generalization performance of the model, short-term forecasts in hours were performed using PM_2.5_, PM_10_, SO_2_, NO_2_, CO, and O_3_ data. The experimental results show that the model proposed in this paper is superior to the comparison model in terms of root mean square error (RMSE) and mean absolute percentage error (MAPE). The MAPE values of the 6 kinds of pollutants are 6.800%, 10.492%, 9.900%, 6.299%, 4.178%, and 7.304%, respectively. Compared with the baseline LSTM and GRU models, the average reduction is 49.111% and 43.212%, respectively.

## Introduction

### Background

Air pollution has long been a major health problem worldwide. According to the World Health Organization (WHO), air pollution directly or indirectly causes 7 million deaths worldwide every year. In China, ${\mathrm{PM}}_{2.5}$, ${\mathrm{PM}}_{10}$,${\mathrm{SO}}_{2}$, ${\mathrm{NO}}_{2}$,$\mathrm{CO and }{\mathrm{O}}_{3}$ are considered the main air pollutants^1^. As the capital of China, Beijing has a permanent resident population of more than 21 million year-round. The high exposure rate of air pollutants brought about by the high population density makes some respiratory diseases, cardiovascular diseases, and allergic diseases more prone to occur^2–4^. At the same time, due to the complexity of its chemical composition, the increase in the concentration of particulate matter in the air will affect the susceptible population from the two aspects of carcinogenicity and mutagenicity^5^. Therefore, developing a model with high prediction accuracy and excellent generalization performance will help the local government adjust air pollution prevention and control strategies in a more reasonable, timely, and targeted manner and improve people's quality of life and health.

### Literature review

At present, there are mainly statistical analysis methods, machine learning methods, and deep learning methods in the prediction of air pollutant concentrations and other atmospheric environment data. Among them, the statistical analysis methods are mainly the linear regression model (LR)^6^ and traditional time series analysis methods, such as the autoregressive moving average model (ARMA)^7^, autoregressive integrated moving average model (ARIMA)^8^ and seasonal autoregressive integrated moving average model (SARIMA)^9^. Some scholars have also used probability distribution and parameter estimation methods to fit wind data and evaluate wind potential. Sharma P K et al.^10^ compared the effect of different probability distribution models on wind data fitting. Gautam et al.^11^ compared 11 Weibull parameter estimation methods and presented the applicable scope, advantages, and disadvantages of each method based on empirical analysis. Some scholars have also made comparisons between heuristic algorithms and numerical methods to estimate wind potential^12^. Although the regression model provides interpretability to a certain extent, its assumption of prior conditions makes it perform poorly in prediction accuracy, while the autoregressive model has great performance in the time series with distinct features, but the fitting ability for complex series is poor. Machine learning models are mainly used for single model prediction and ensemble model prediction, such as support vector regression (SVR)^13^ and random forest regression (RFR)^14^.

With the development of deep learning and the improvement of computer computing power, neural network models with excellent fitting ability to time series are used by scholars to predict the concentration of air pollutants. Among them, the representative model of the recurrent neural network (RNN) is the long short-term memory network (LSTM)^15,16^, and the gated recurrent unit network (GRU)^17^ has been verified to outperform ARMA-type models in air pollutant concentration prediction. After He et al.^18^ proposed the residual network, the effectiveness of using the convolution method to extract series features is guaranteed, and the attention mechanism proposed by Tom et al.^19^ has also been verified by scholars to effectively improve the prediction accuracy of the model by emphasizing the important moments^20^. Combined with the research of previous scholars, the convolution-RNN network model based on the attention mechanism has been applied to the field of time series prediction, and models such as convolutional neural networks-gate recurrent unit-attention network (CNN-GRU-Attention)^21^ and convolutional neural networks-long short-term memory-squeeze-and-excitation network (CNN-LSTM-SENet)^22^ have been developed and have achieved good prediction accuracy. It should be noted that the prediction accuracy of the neural network model is very dependent on the nature of the series data itself, so good data preprocessing can improve the learning and generalization capabilities of the network to a certain extent. Among them, seasonal and trend decomposition using locally weighted scatterplot smoothing (Loess) (STL)^23^ using robust locally weighted regression as a smoothing method has been widely used in time series preprocessing^24^. Jiao et al.^25^ combined it with a neural network model to obtain STL-LSTM for bus passenger flow prediction during the COVID-19 epidemic.

**Figure 1:**
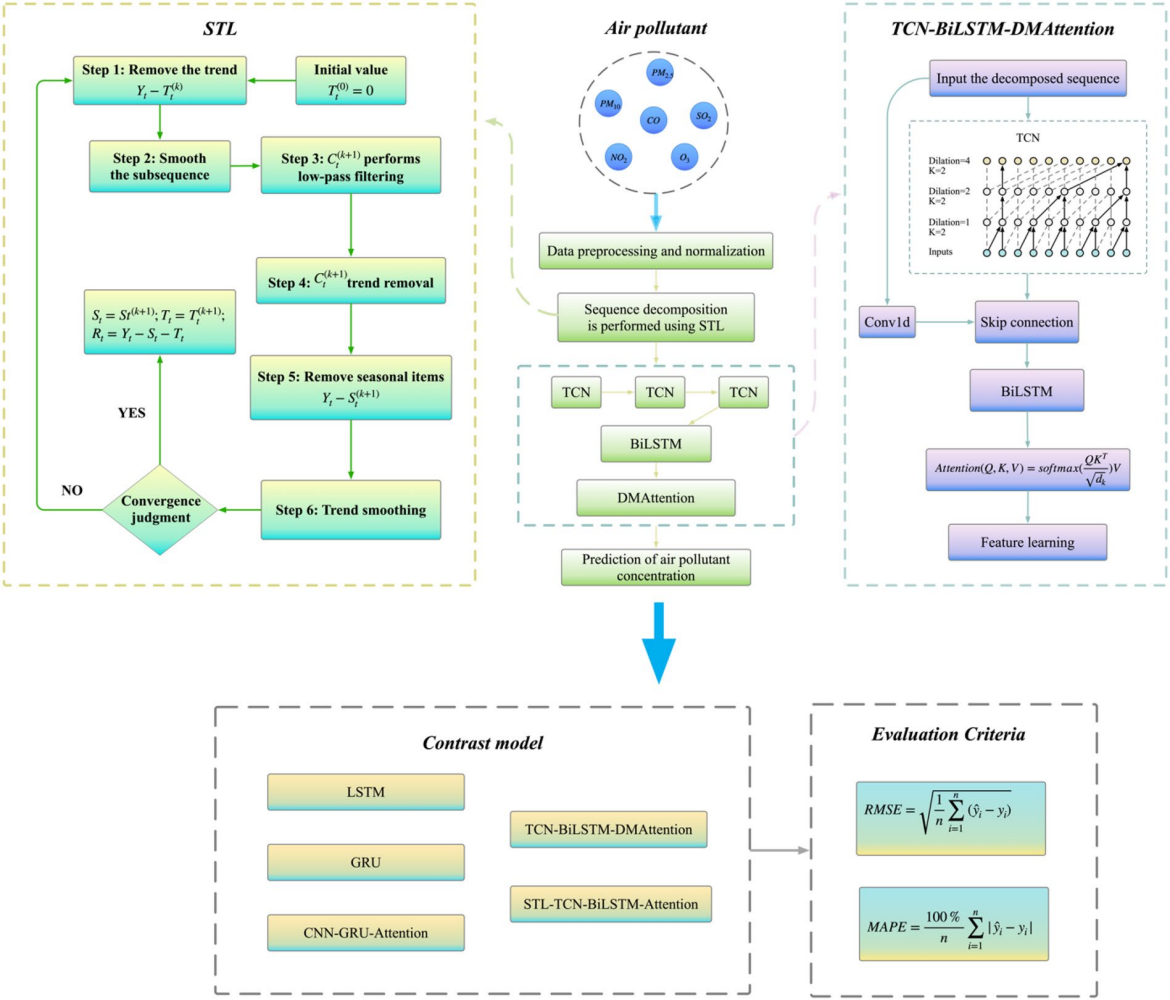
Flowchart of this paper.

Combining the previous research results, this paper proposes a network structure based on STL for series decomposition and temporal convolution (TCN) and a bidirectional long short-term memory network (BiLSTM) for series feature learning after decomposition. Inspired by the research of Fu et al.^26^, a dependency matrix attention mechanism (DMAttention) based on cosine similarity is proposed. In this paper, the series processed by STL-TCN-BiLSTM is input to DMAttention for feature emphasis at similar moments, which is used for short-term concentration prediction of various air pollutants. This paper uses the air quality detection data of 12 stations established by the China Environmental Monitoring Station in Beijing to analyze the accuracy of model prediction with LSTM and GRU as the baseline model and simultaneously conducts comparative ablation experiments to verify the validity of each module of the model proposed in this paper. The flowchart of this paper is shown in Fig. 1.

### Main contribution

The main contribution of the article is as follows:

1. Proposing a network structure for feature learning of time series. Experiments show that the structure has excellent accuracy in predicting air pollutant concentrations.
2. Proposing a dependency matrix attention mechanism based on cosine similarity. This module plays a positive role in improving the prediction accuracy and is superior to the ordinary attention mechanism in predicting the concentration of air pollutants.
3. The proposed model achieved good results in prediction accuracy. Compared with LSTM, GRU, and their combination models, the model proposed in this paper is more accurate in predicting air pollutant concentrations.
4. Excellent generalization properties have been achieved for different pollutants. In the prediction of six kinds of air pollutants, the model proposed in this paper achieved good prediction accuracy.

## Research methods

### STL

STL decomposes the time series into trend items, seasonal items, and residual items in the form of an additive model through locally weighted regression, which is robust and less affected by series outliers.

1 $$ Y_{t} = T_{t} + S_{t} + R_{t} $$

In Eq. (1), ${Y}_{t}$, ${T}_{t}$, ${S}_{t}$, and ${R}_{t}$ represent the time series observations at time $\mathrm{t}$ and their corresponding trend items, seasonal items, and residual items, respectively. The pseudocode of STL decomposition is listed in Algorithm 1.
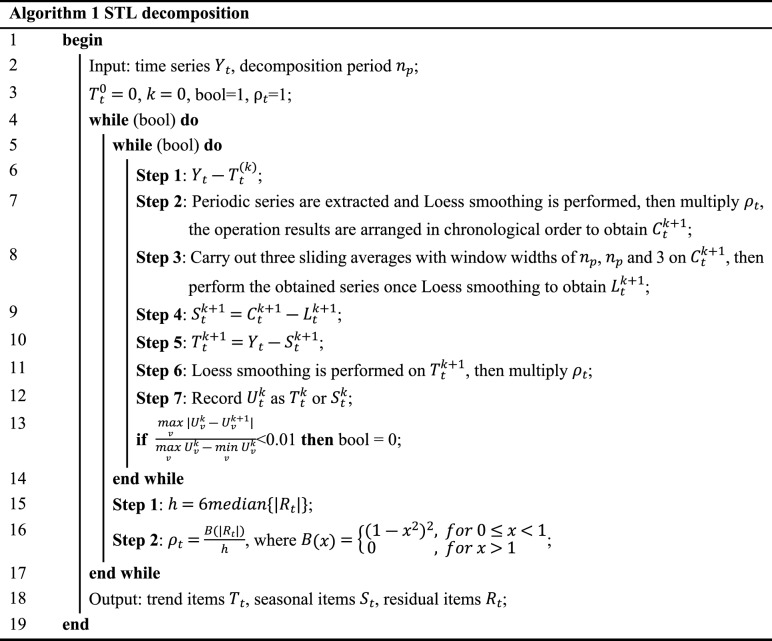


The STL method obtains ${T}_{t}$ and ${S}_{t}$ through the inner loop iteration and obtains ${R}_{t}$ through the additive model. To reduce the impact of outliers in the series, a robust weight ${\uprho }_{t}$ is introduced in the outer loop. During the Loess smoothing process of inner loop iterations Step 2 and Step 6, the adjacency matrix is multiplied by ${\uprho }_{t}$ to constrain the outliers to improve the robustness of series decomposition.

### TCN

TCN is mainly composed of causal dilated convolution and residual block. This method can effectively avoid vanishing and exploding gradients while performing time series feature extraction.

1. Causal dilated convolution.

The causal convolution in the TCN can be expressed as the value at moment t of the layer h network only depending on the value at moment t and its previous moment in layer h−1. The difference between the causal convolution structure and the traditional convolution is that the convolution area is strictly limited, which is reflected in the time constraint in the time series; that is, the value of the future moment is not considered in the feature extraction process.

**Figure 2:**
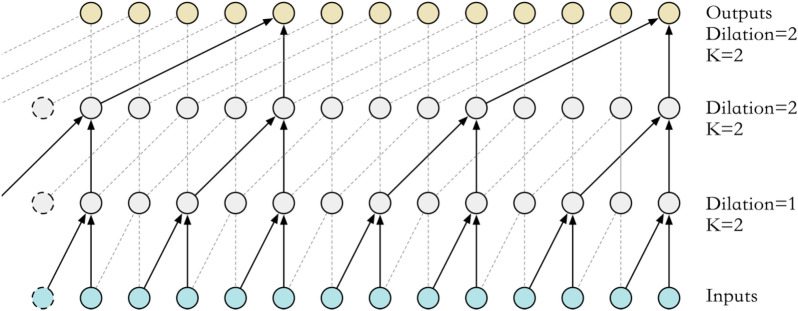
Causal dilated convolutional structure.

As shown in Fig. 2, interval sampling is performed on the basis of causal convolution to increase the receptive field. Compared with multilayer convolution and pooling to increase the receptive field, this method can effectively reduce information loss and does not use unit convolution kernels; at the same time, the input and output time steps are the same. The operation of causal dilated convolution is shown in Eq. (2).

2 $${y}_{h,t}=\sum_{i=0}^{k}{f}_{i}\cdot {y}_{h-1,t-2id}$$

where ${y}_{h,t}$ represents the series value of layer $h$ in the network at time $t$, ${f}_{i}$ represents the filter, *k* represents the size of the convolution kernel, and $d$ represents the convolutional dilation rate.

2. Residual block.

The skip connection enables the network to carry out cross-layer information transfer to avoid the problem of vanishing and exploding gradients in the network. The structure is shown in Fig. 2.

**Figure 3:**
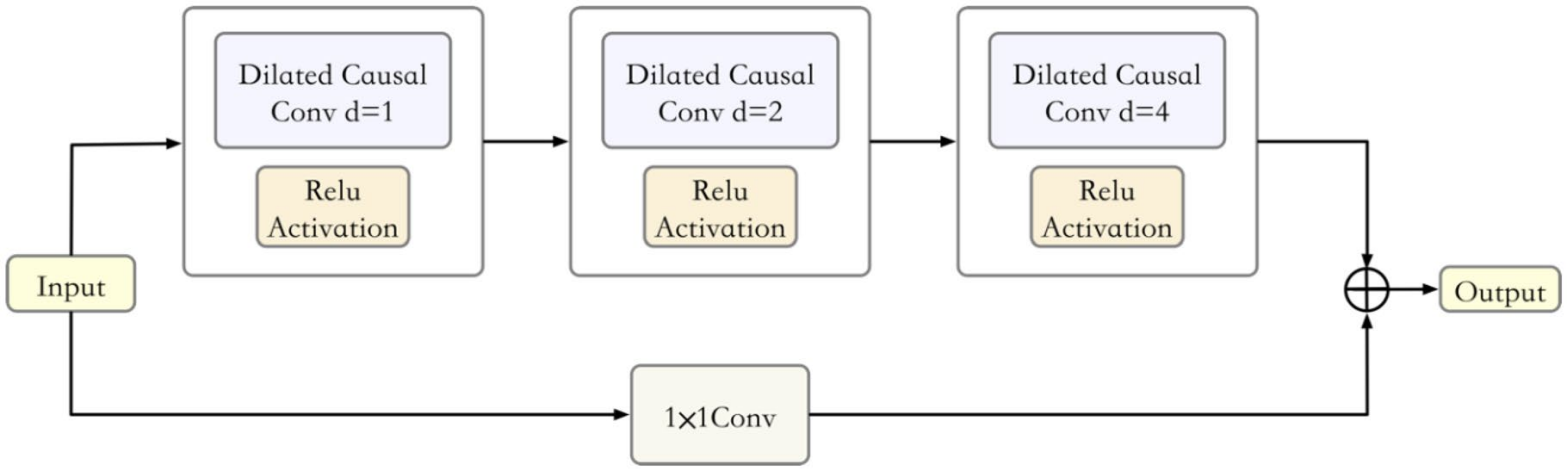
Residual block structure.

As shown in Fig. 3, the TCN set up in this paper performs three causal dilated convolutions, and the dilation rates are 1, 2, and 4. The unit convolution kernel is used to process the input so that the series structure of the two paths is the same for the summation operation.

### BiLSTM

**Figure 4:**
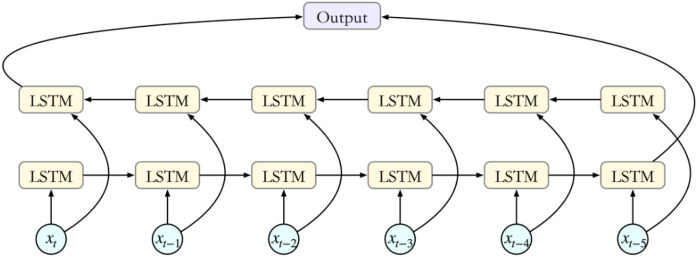
BiLSTM structure.

LSTM adjusts the information flow by setting the gate structure, retains important information in the series learning process, and forgets secondary information to achieve the effect of "long-term memory". BiLSTM is a bidirectional LSTM that inputs the time series into the LSTM model in the forward and reverse directions for feature extraction and stitches the results to obtain more feature information. The structure is shown in Fig. 4.

### DMAttention

Inspired by DANet, this paper proposes a cosine similarity-based dependency matrix attention mechanism (dependency matrix attention) for multivariate time series forecasting. The structure is shown in Fig. 4.

**Figure 5:**
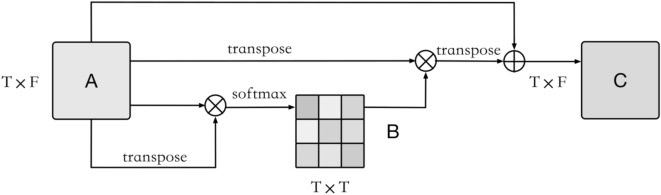
DMAttention structure.

As shown in Fig. 5, DMAttention first performs an inner product operation on the eigenvectors at each moment, that is, $\mathrm{A}\cdot {\mathrm{A}}^{\mathrm{T}}$, and then performs a $softmax$ operation to obtain a weight matrix $B$ that reflects the dependencies at each moment, as shown in Eq. (3).

3 $${x}_{ij}=\frac{\mathrm{exp}\left({A}_{i,}^{T}\cdot {A}_{j}\right)}{\sum_{i=1}^{T}\mathrm{exp}\left({A}_{i,}^{T}\cdot {A}_{j}\right)}$$

${x}_{ij}$ represents the dependency relationship between moment $i$ and moment $j.$ If the eigenvectors of the two moments have a high similarity, their corresponding weights will also be relatively high. Then, matrix multiplication of ${\mathrm{A}}^{\mathrm{T}}$ and $B$ is performed and transposed to obtain the weighted sum of the features at each moment and finally multiply the scale coefficient $\upgamma$ that changes with the iteration and perform a skip connection to obtain the output matrix $C$, as shown in Eq. (4).

4 $$C=\gamma {\left({A}^{T}\cdot B\right)}^{T}+A$$

DMAttention can assign higher weights to eigenvectors with higher similarity regardless of the time interval, highlight important moments, and enhance feature representation. It has achieved better results in air pollutant concentration prediction than the attention mechanism based on full connection.

### STL-TCN-BiLSTM-DMAttention

**Figure 6:**
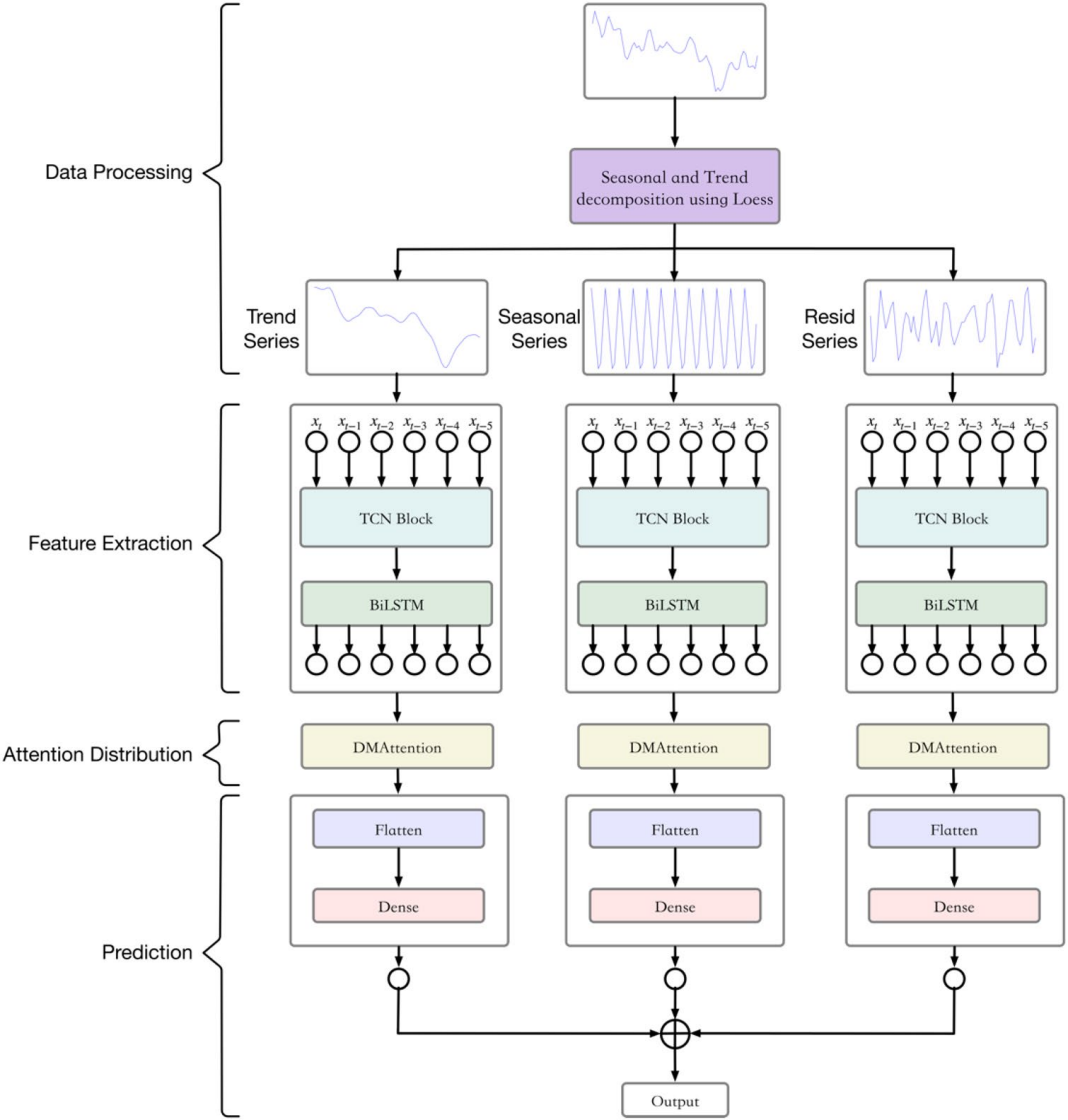
STL-TCN-BiLSTM-DMAttention structure.

The STL-TCN-BiLSTM-DMAttention model consists of four parts: data preprocessing, data feature extraction, attention allocation, and air pollutant concentration prediction. The structure is shown in Fig. 6.

As shown in Fig. 6, the model first decomposes the series in terms of trend, season, and remainder according to the specified period, inputs the three subseries into the TCN-BiLSTM layer for feature extraction and analysis, enters the DMAttention layer for attention allocation and finally performs the series flattening and full connection operations. The sum of the obtained subseries is the final prediction result.

## Empirical analysis

### Data sources

The experimental data used in this paper came from the real-time environmental detection data of 12 detection points of China’s environmental monitoring stations in Beijing (http://www.cnemc.cn). The data recording interval is hours and has been averaged, starting from 0:00 on January 1, 2018, to 24:00 on April 30, 2022, for a total of 37,008 samples, each sample recorded as ${\mathrm{PM}}_{2.5}$, ${\mathrm{PM}}_{10}$, ${\mathrm{SO}}_{2}$, ${\mathrm{NO}}_{2}$, and $\mathrm{CO and }{\mathrm{O}}_{3}$. This paper uses the designed model to predict the concentration of 6 pollutants at time $t+1$ separately based on the information from the previous $t$ moments.

### Data preprocessing

There are 585 missing values in ${\mathrm{PM}}_{10}$ in the original data set and 1 missing value in ${\mathrm{PM}}_{2.5}$. For the missing part, this paper uses the mean value before and after the missing value to fill. To reduce the influence of extreme values on the model and improve the convergence rate, this paper normalizes the deviation of each column feature.

### Experimental design

In this paper, the data set was divided into a training set, verification set and test set at a ratio of 6:2:2 in time order. After comprehensive consideration of calculation cost and prediction accuracy, the data of 6 moments were used as model input to predict the pollutant concentration at the latter moment.

#### Main hyperparameter settings

The hyperparameters used in this model are shown in Table 1.

**Table 1:** Parameter settings for the proposed model.

Item	Hyperparameter type	Value
Seasonal and Trend decomposition using Loess	Seasonal periodicity	6
Causal dilated conv layer 1	Dilation rate	1
	Convolution kernels	32
	Kernel size	3
Causal dilated conv layer 2	Dilation rate	2
	Convolution kernels	32
	Kernel size	3
Causal dilated conv layer 3	Dilation rate	4
	Convolution kernels	16
	Kernel size	3
BiLSTM	Each direction unit size	4
Optimizer	Adam	Default

#### Evaluation criteria

In this paper, the root mean square error (RMSE) and mean absolute percentage error (MAPE) are used to evaluate the prediction results of the model. The smaller the RMSE and MAPE were, the better the prediction effect of the model was.

The calculation formula of RMSE is shown in Eq. (5).

5 $$RMSE=\sqrt{\frac{1}{n}\sum_{i=1}^{n}{\left(\widehat{{y}_{i}}-{y}_{i}\right)}^{2}}$$

The calculation formula of MAPE is shown in Eq. (6).

6 $$MAPE=\frac{100\%}{n}\sum_{i=1}^{n}\left|\widehat{{y}_{i}}-{y}_{i}\right|$$

where $\widehat{{y}_{i}}$ represents the predicted value and ${y}_{i}$ represents the true value.

#### Model prediction results

**Figure 7:**
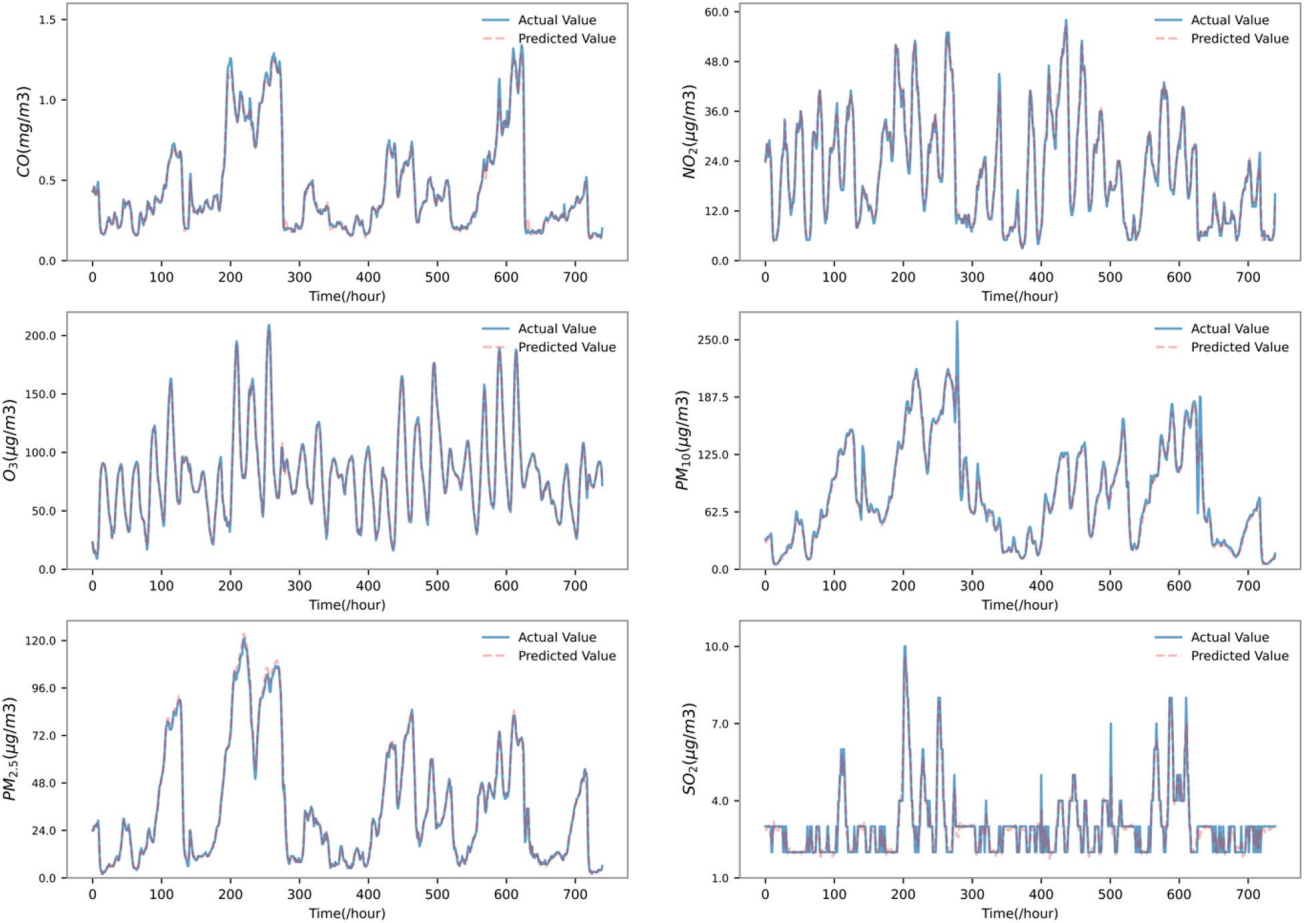
Forecast results of 6 kinds of pollutants.

In this paper, according to the above experimental settings, short-term predictions are made on ${\mathrm{PM}}_{2.5}$, ${\mathrm{PM}}_{10}$, ${\mathrm{SO}}_{2}$, ${\mathrm{NO}}_{2}$, and $\mathrm{CO and }{\mathrm{O}}_{3}$, and a visual comparison with the real values is shown in Fig. 7 (due to the large sample size of the test set, only the last 1 month is used for demonstration).

As shown in Fig. 7, STL-TCN-BiLSTM-DMAttention has a good prediction effect on the 6 pollutants, and the calculated RMSE and MAPE values are shown in Table 2.

**Table 2:** Measurement of the prediction accuracy of the proposed model for 6 air pollutants.

Air pollutant	${\mathrm{PM}}_{2.5}$	${\mathrm{PM}}_{10}$	${\mathrm{SO}}_{2}$	${\mathrm{NO}}_{2}$	$\mathrm{CO}$	${\mathrm{O}}_{3}$
RMSE	1.973	6.054	0.437	1.545	0.076	2.922
Air pollutant	${\mathrm{PM}}_{2.5}$	${\mathrm{PM}}_{10}$	${\mathrm{SO}}_{2}$	${\mathrm{NO}}_{2}$	$\mathrm{CO}$	${\mathrm{O}}_{3}$
MAPE	6.800%	10.492%	9.900%	6.299%	4.178%	7.304%

According to the results of the two evaluation indicators, the model has good prediction accuracy in the prediction of air pollutant concentrations.

#### Comparative analysis of models

To test the effectiveness of each module of the STL-TCN-BiLSTM-DMAttention proposed in this paper and the overall high prediction accuracy of the model, the following four comparative experiments are designed in this paper.

1. STL-TCN-BiLSTM-attention.

The DMAttention module of the proposed model is replaced with fully connected attention to verify the effectiveness of DMAttention in air pollutant prediction.

2. TCN-BiLSTM-DMAttention.

The sequence decomposition module is removed, and experiments are conducted to verify the effectiveness of STL decomposition.

3. CNN-GRU-attention.

We use the CNN-GRU-attention model, which has a similar structure, to conduct experiments and compare it with the model proposed in this paper to verify the effectiveness of the overall structure of the model.

4. LSTM, GRU

In this paper, LSTM and GRU are set as baseline models to compare with the proposed model to verify that the model has high prediction accuracy.

**Figure 8:**
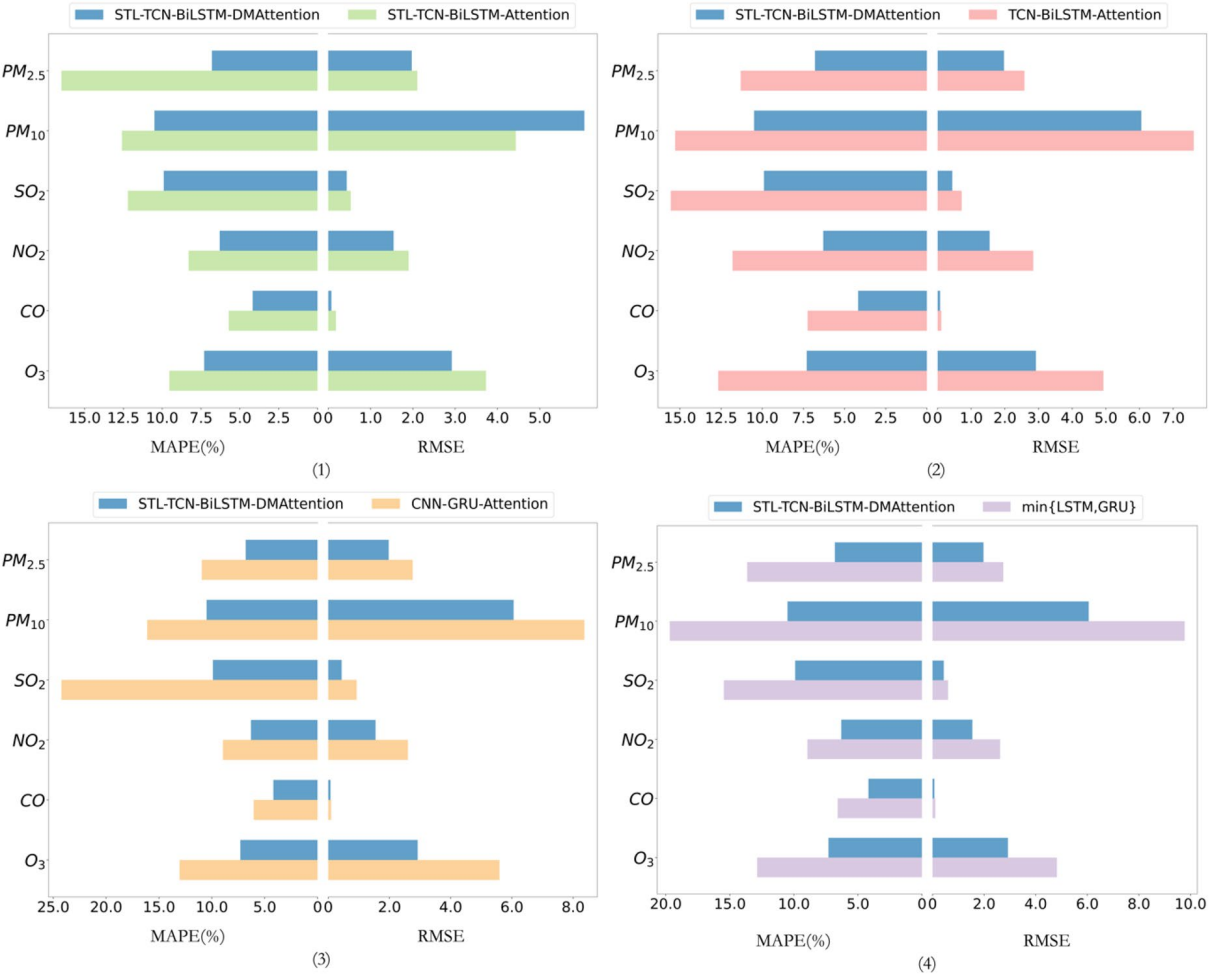
The results of comparative experiments.

The data were used to obtain the results of the above four comparative experiments, as shown in Fig. 8.

Combined with Fig. 8 and Table 3, it can be obtained that (1) the MAPE is reduced by an average of 30.644% when using the DMAttention module compared to the fully connected attention module. The overall model is better than STL-TCN-BiLSTM-attention, and the prediction accuracy is higher. (2) Using STL for trend decomposition reduces the MAPE of the model by an average of 39.136%. (3) Compared with the CNN-GRU-attention network with the same convolution-RNN-attention structure, the MAPE is reduced by an average of 43.319%. (4) Compared with the baseline LSTM model, MAPE is reduced by an average of 43.212%, and compared with GRU, MAPE is reduced by an average of 49.111%. Comparing the model proposed in this paper with the optimal results of LSTM and GRU, as shown in Fig. 8, the accuracy of the concentration prediction of the 6 air pollutants is higher.

**Table 3:** Forecast error of different models.

Air pollutant	LSTM		GRU		CNN-GRU-Attention		TCN-BiLSTM-DMAttention		STL-TCN-BiLSTM-Attention		STL-TCN-BiLSTM-DMAttention	
	RMSE	MAPE (%)	RMSE	MAPE (%)	RMSE	MAPE (%)	RMSE	MAPE (%)	RMSE	MAPE (%)	RMSE	MAPE (%)
${\mathrm{PM}}_{2.5}$	2.813	14.701	2.743	13.646	2.760	10.954	2.581	11.313	2.106	16.489	1.973	6.800
${\mathrm{PM}}_{10}$	9.772	19.685	10.541	21.701	8.367	16.125	7.611	15.287	4.432	12.593	6.054	10.492
${\mathrm{SO}}_{2}$	0.659	18.841	0.604	15.472	0.930	24.230	0.715	15.559	0.533	12.208	0.436	9.900
${\mathrm{NO}}_{2}$	2.698	10.830	2.620	8.938	2.605	8.950	2.843	11.805	1.901	8.299	1.545	6.299
CO	0.113	7.742	0.110	6.575	0.094	6.044	0.108	7.250	0.183	5.718	0.076	4.178
${\mathrm{O}}_{3}$	5.106	16.581	4.820	12.868	5.592	13.049	4.926	12.680	3.729	9.538	2.922	7.304
Average	–	14.730	–	13.200	–	13.225	–	12.316	–	10.808	–	7.496

The concentration of air pollutants is often related to humidity, temperature, light, air convection, and other human activities^27,28^. In this paper, STL is used to perform robust trend and seasonal decomposition of the original series, revealing its seasonal and persistent characteristics. The neural network model for well-processed series has a stronger learning ability and obtains more accurate prediction results. The decomposed series information is input into the TCN for interval sampling to obtain a larger receptive field, which can be better recognized by BiLSTM. The bidirectional LSTM network structure is better placed to make good use of the input feature information and then emphasize the moment with higher dependency through DMAttention. The experimental results show that the proposed model is more accurate than the other five models in predicting the concentrations of 6 kinds of air pollutants.

## Conclusions

Considering that hourly air pollution concentration data are susceptible to the influence of light and temperature, an STL-TCN-BiLSTM-DMAttention model is proposed to present a short-term concentration prediction. The STL method is used to obtain trend and seasonal information, and the TCN-BiLSTM structure is used to fully extract the series characteristics of trend, season, and remaining information through causal dilated convolution and bidirectional time series information processing. Finally, based on the DMAttention mechanism proposed in this paper, the moment with a high degree of global dependency is captured, and the final prediction result is obtained by flattening and fully connected layers.

To verify the accuracy and generalization performance of the proposed model, this paper sets LSTM and GRU as the baseline model, deletes and replaces each module of the model and compares it with the CNN-GRU-attention model, which has a similar structure in 6 kinds of air pollutant concentration prediction accuracy. According to the experimental results, the following conclusions are drawn: (1) STL decomposition can effectively extract the hourly trend and seasonal characteristics of air pollutant concentrations, which greatly improves the prediction accuracy of the model. (2) The TCN-BiLSTM module performs well in feature learning of decomposed series. (3) The DMAttention proposed based on the cosine similarity can capture the information required by the model better than the attention mechanism that obtains the weight vector through the fully connected layer in the prediction of the air pollutant concentration. (4) The proposed STL-TCN-BiLSTM-DMAttention model has an average reduction of 43.212% and 49.111% on MAPE compared to LSTM and GRU, respectively. (5) For the concentrations of 6 common air pollutants, the models proposed in this paper have achieved excellent prediction results, and the models have strong generalization performance.

However, this paper still notes that some aspects are worthy of further research, such as (1) Adding natural factors such as air temperature, solar radiation and air convection and human factors such as traffic flow and gas usage to the model for prediction. (2) Extending the short-term forecast to the long-term forecast by adjusting the model structure and modules. (3) Using more diverse data sets to explore the application value of the model in other fields.

## Data Availability

The data sets used and/or analyzed during the current study are available from the corresponding author on reasonable request.

## Appendix

**Table:** Appendix 1 Main abbreviations and Greek letters with their explanations.

Abbreviation	Explanation
RNN	Recurrent neural network
LSTM	Long short-term memory network
GRU	Gated recurrent unit network
STL	Seasonal and trend decomposition using locally weighted regression
TCN	Temporal convolutional network
BiLSTM	Bidirectional long short-term memory network
DMAttention	Dependency matrix attention
RMSE	Root mean square error
MAPE	Mean absolute percentage error
Loess	Locally weighted scatterplot smoothing

Greek letter	Explanation
${Y}_{t}$	Observations at time t
${T}_{t}$	Trend items at time t
${S}_{t}$	Seasonal items at time t
${R}_{t}$	Residual items at time t
${n}_{p}$	Decomposition period
${\rho }_{t}$	Robust weight
${C}_{t}^{k}$	Intermediate variable which is performed Loess
${L}_{t}^{k}$	Intermediate variable that Loess according to the specified period
${y}_{h, t}$	The series value of layer $h$ in the network at time $t$
${f}_{i}$	Filter in temporal convolutional network
$A$	One period of data
$B$	Dependency matrix of a period of data
$C$	A period of data processed by dependency matrix attention
